# The *Aspergillus fumigatus* cell wall integrity signaling pathway: drug target, compensatory pathways, and virulence

**DOI:** 10.3389/fmicb.2015.00325

**Published:** 2015-04-16

**Authors:** Vito Valiante, Juliane Macheleidt, Martin Föge, Axel A. Brakhage

**Affiliations:** ^1^Molecular Biotechnology of Natural Products, Department of Molecular and Applied Microbiology, Leibniz Institute for Natural Product Research and Infection Biology – Hans Knöll InstituteJena, Germany; ^2^Department of Microbiology and Molecular Biology, Institute of Microbiology, Friedrich Schiller UniversityJena, Germany

**Keywords:** *Aspergillus fumigatus*, cell wall integrity, signaling pathways, virulence, mitogen activated protein kinases (MAPKs)

## Abstract

*Aspergillus fumigatus* is the most important airborne fungal pathogen, causing severe infections with invasive growth in immunocompromised patients. The fungal cell wall (CW) prevents the cell from lysing and protects the fungus against environmental stress conditions. Because it is absent in humans and because of its essentiality, the fungal CW is a promising target for antifungal drugs. Nowadays, compounds acting on the CW, i.e., echinocandin derivatives, are used to treat *A. fumigatus* infections. However, studies demonstrating the clinical effectiveness of echinocandins in comparison with antifungals currently recommended for first-line treatment of invasive aspergillosis are still lacking. Therefore, it is important to elucidate CW biosynthesis pathways and their signal transduction cascades, which potentially compensate the inhibition caused by CW- perturbing compounds. Like in other fungi, the central core of the cell wall integrity (CWI) signaling pathway in *A. fumigatus* is composed of three mitogen activated protein kinases. Deletion of these genes resulted in severely enhanced sensitivity of the mutants against CW-disturbing compounds and in drastic alterations of the fungal morphology. Additionally, several cross-talk interactions between the CWI pathways and other signaling pathways are emerging, raising the question about their role in the CW compensatory mechanisms. In this review we focused on recent advances in understanding the CWI signaling pathway in *A. fumigatus* and its role during drug stress response and virulence.

## Overview About Cell Wall Organization in *Aspergillus fumigatus*

The fungal cell wall (CW) is the exoskeleton of fungal cells. Besides its starkness and firmness, it has a very flexible structure, which, therefore, can alter rapidly and efficiently in response to external and internal stimuli. In particular, filamentous fungi adapt their internal pressure and turgor in concert with CW-biosynthesis enzymes, in order to direct hyphal growth, following gradients of nutrients, and chemo attractants (e.g., hormones), or to avoid adverse habitats (Brand and Gow, 2009; Lew, 2011).

In recent years, the composition of the fungal CW was extensively studied. It varies among different species but is mostly composed of polymers of sugars, which show a high degree of branching. Although the CW composition varies among different fungal species, there are conserved parts such as a common core composed of branched β-1,3-glucan-chitin. An exception was found in zygomycetes, which contain chitosan instead of chitin (Singh et al., 2011). The polysaccharide-based three-dimensional network in *Aspergillus fumigatus* is completed by the addition of sugar-chains composed of α-1,3-glucan, galactofuran, and mannan, which make the structure rather variable compared to other fungi (Latge and Beauvais, 2014). Besides the polysaccharide structure, the CW is adorned with a variety of proteins. In *A. fumigatus* dormant conidia, the cell surface is covered by a rodlet layer, which is composed of regularly arranged RodA hydrophobin proteins. This hydrophobin envelope was found to be essential to immunologically mask the conidia, which are continuously inhaled by humans (Aimanianda et al., 2009). Other hydrophobins, which are produced at different developmental stages in *A. fumigatus*, were associated with drug response (Gautam et al., 2008) and biofilm-growth conditions (Bruns et al., 2010).

In the CW biosynthesis, the β-1,3-glucan synthase (Fks1) plays the major role (Beauvais et al., 2001). Fks1 is an integral plasma membrane protein having 16 putative *trans*-membrane helices. The genome of the majority of fungi normally contains only a single β-1,3-glucan synthase gene. Consequently, the deletion of this gene was supposed to be lethal (Firon et al., 2002; Henry et al., 2012). However, a recent publication reported the characterisation of an *A. fumigatus* Δ*fks1* mutant, which appeared to be viable besides showing a severe growth phenotype (Dichtl et al., 2015).

The *A. fumigatus* genome harbors many putative genes responsible for CW modifications. Different β-glucanases and branching enzymes were identified (Mouyna et al., 2013). These genes are more difficult to analyze by classical reverse genetics, because some of them are apparently functionally redundant. An example is given by the β-1,3-glucanosyltransferase (Gel) family, which, in *A. fumigatus*, is composed of seven different members. Among them, only the deletion of *gel4* was reported to be lethal (Gastebois et al., 2010), whereas the Δ*gel2*, Δ*gel1*, and Δ*gel7* deletion mutant strains were viable (Mouyna et al., 2005; Zhao et al., 2014).

For chitin, which is the second most abundant polysaccharide of the CW, a very similar situation was found. Among the eight putative chitin synthase genes identified in *A. fumigatus*, only the deletion of *csmA* and *csmB* led to a significantly altered phenotype, i.e., reduction of the colony radial growth rate and decrease in chitin content (Aufauvre-Brown et al., 1997; Jimenez-Ortigosa et al., 2012), while the mutation of the remaining genes did not result in a significant phenotype, and obvious phenotypical changes of mutants were only reported upon multiple simultaneous gene disruptions (Rogg et al., 2011; Muszkieta et al., 2014).

The knowledge about genes involved in the biosynthesis of the CW is steadily increasing. In total, more than 30% of the *A. fumigatus* genome still encodes genes with unknown function (Cerqueira et al., 2014). Thus, it is reasonable to assume that in the future further enzymes involved in CW biosynthesis will be discovered, likely having novel enzymatic functions. As an example, it was predicted that *A. fumigatus* potentially expresses more than 100 different glycosylphosphatidylinositol (GPI) anchored proteins (Cao et al., 2009), which are likely to form a bridge between the membrane lipid bilayer and the CW. The majority of these proteins are supposed to play a role in the CW formation, but only very few have been characterized so far (Li et al., 2007).

## Activity of Antifungal Drugs Targeting the Fungal Cell Wall and Resistance Mechanisms

From all the different classes of potential antifungal drugs, only three of them have a wide clinical use against invasive and systemic infections caused by *A. fumigatus*. These compounds belong to the polyenes, azoles, and echinocandins (Ullmann and Cornely, 2006).

**FIGURE 1:**
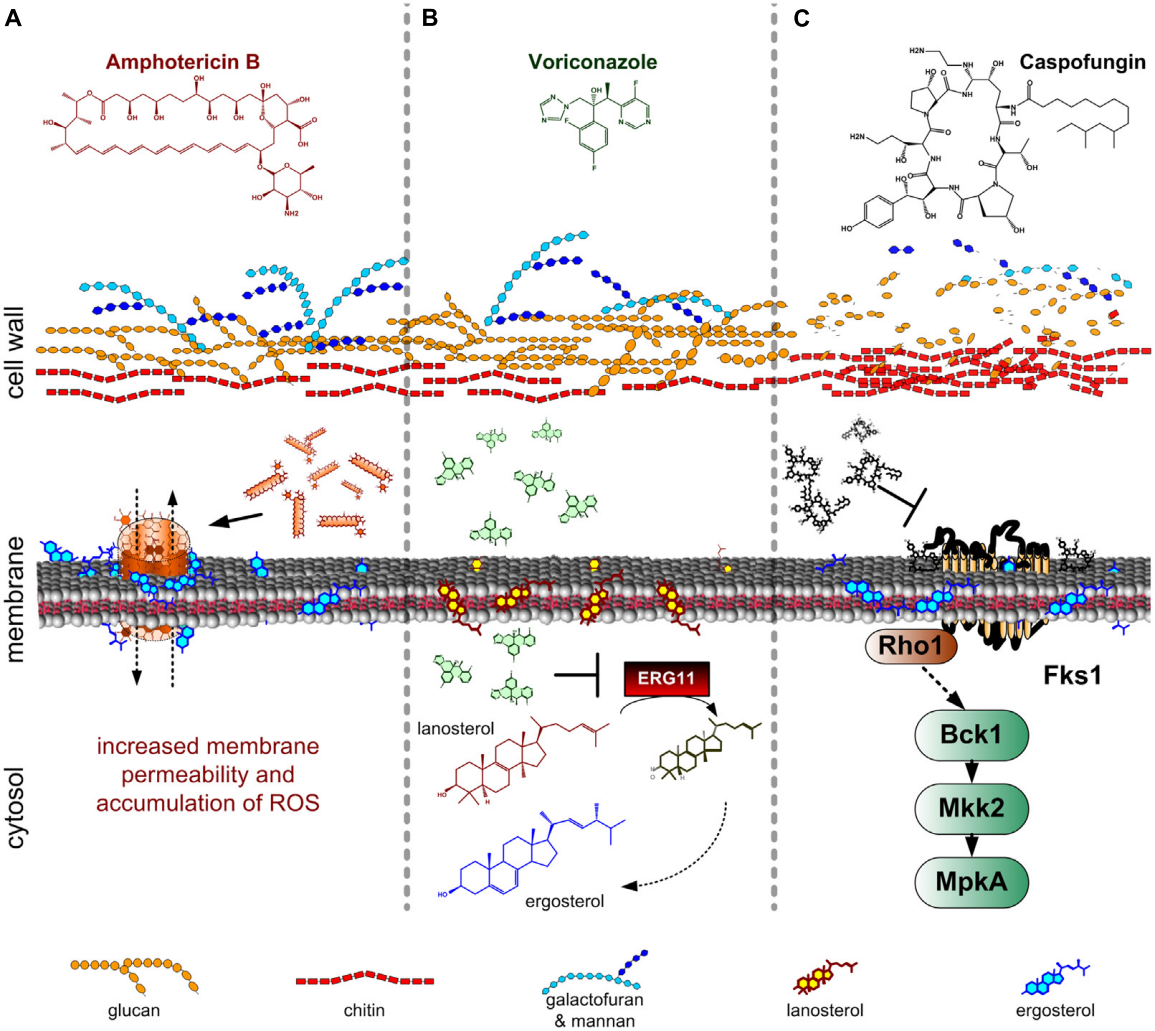
**Mode of actions of antifungal drugs commonly used against invasive and systemic *Aspergillus fumigatus* infection (Ullmann and Cornely, 2006). (A)** Polyenes (in this case amphotericin **B**) bind to sterols, forming pores in the cell membrane. The formation of these pores leads to increased membrane permeability. Additionally, amphotericin B induces the accumulation of reactive oxygen species (ROS), which have multiple toxic effects on fungal cells; **(B)** Azoles (in this case voriconazole) inhibit the ERG11 enzyme thereby blocking the ergosterol biosynthesis. ERG11 catalyses the formation of 4,4-dimethylcholesta-8,14,24-trienol from lanosterol. The lack of ergosterol and the subsequent accumulation of lanosterol, results in high toxicity for the cell; **(C)** Echinocandins (in this case caspofungin) inhibit the β-1,3-glucan synthesis. As a response to the reduction of glucan content there is an increase in chitin biosynthesis.

Polyenes and azoles are mainly targeting ergosterol and its biosynthesis, respectively, while echinocandins specifically inhibit β-1,3-glucan formation (**Figure 1**). However, it is increasingly acknowledged that defense mechanisms against these drugs have common elements. Consistently, a genome-wide screen conducted in *Schizosaccharomyces pombe* indicated that different ergosterol biosynthesis deficient mutants were also sensitive to β-1,3-glucan synthase inhibitor echinocandins and β-glucanase (Fang et al., 2012). A similar observation was made for *Candida albicans* biofilms, which normally exhibit higher resistance to polyenes depending on ergosterol and β-1,6-glucan synthesis (Khot et al., 2006).

Polyenes belong to the oldest known antifungal compounds. Amphothericin B (AmB), first isolated from *Streptomyces nodosus*, is the most commonly used polyene against systemic infections (Blum et al., 2013). AmB possesses a name-giving amphoteric character, which allows the binding of the lipophilic compound to the cytoplasmic membrane. The mode of action of polyenes is not fully understood. They theoretically bind to all kinds of sterols, with a significant higher affinity to ergosterol (Bolard, 1986; **Figure 1A**). The fungicidal activity of AmB is potentially based on the formation of channels in the cell membrane, which lead to increased membrane permeability and leakage of small molecules, inhibition of aerobic and anaerobic respiration, and accumulation of reactive oxygen species (ROS) (Mesa-Arango et al., 2014; **Figure 1A**). It was suggested that eight AmB molecules are assembled to a ring-like structure, showing both a hydrophobic interaction to ergosterol with its conjugated double-bond system and forming a hydrophilic pore with its inward directed hydroxyl groups (Baginski et al., 1997). Thus, the sensitivity toward polyenes is dependent on the composition of membranes, with regard to sterol content, and chemical structure of phospholipid fatty acyl chains.

Surprisingly, despite the long-term use of polyenes, resistance to AmB and others polyene drugs remains rare. Nevertheless, there are resistant species of *Candida* (*C. glabrata*, *C. tropicalis*, *C. lusitaniae*), which are characterized by an intrinsically low ergosterol content (Young et al., 2003; Vandeputte et al., 2008; Gray et al., 2012; Eddouzi et al., 2013). The resistance to AmB in *Aspergillus terreus* was attributed to a higher level of catalase expression that counteracts the AmB-induced formation of ROS (Blum et al., 2013). Additionally, resistant environmental *Aspergillus flavus* strains were isolated, which were characterized by an alteration of CW composition and higher levels of α-1,3-glucan (Seo et al., 1999).

The group of azoles comprise a plethora of synthetic compounds, characterized by the presence of either a 2-N-containing imidazole ring or a 3-N-containing triazole ring with complex side-chains of at least one halogenated phenyl group (Bruggemann et al., 2009). In general, azoles show a broad spectrum of antifungal activity, a good toleration by humans and a relative low cytotoxicity, making them the major agents for treatment, and prevention of *Aspergillus* infections. There are many licensed azole antifungal drugs, but only four, namely fluconazole, itraconazole, posaconazole, and voriconazole, are mainly used for treatment of fungal infections (Bruggemann et al., 2009). They all act by inhibiting the fungal cytochrome P450 14α-sterol demethylase, which leads to a decrease in ergosterol in the fungal cytoplasmic membrane and thus to inhibition of growth (**Figure 1B**). In *A. fumigatus*, two genes have been assigned to this enzymatic function: *cyp51A* and *cyp51B* (*erg11*) (Mellado et al., 2001). The deduced proteins catalyze the oxidative removal of the 14α-methyl group of lanosterol or eburicol, respectively, (**Figure 1B**). However, single deletions of genes involved in either demethylation or desaturation of ergosterol intermediates such as *cyp51A*, *cyp51B*, and *erg3A*, *erg3B* and *erg3C*, respectively, showed differences in relative sterol composition, but further tests indicated that neither of these genes is individually essential for *A. fumigatus* survival and virulence (Mellado et al., 2005; Hu et al., 2007; Alcazar-Fuoli and Mellado, 2012).

The main mechanism of resistance of *A. fumigatus* against azole antifungal drugs is based on a mutation of the *cyp51A* gene locus with a conversion of leucine at position 98 into histidine (L98H) in combination with the appearance of a 34-bp tandem repeat in *cyp51A* promoter region (TR_34_). In addition to TR34/L98H, also other hot spots for point mutations in the *cyp51A* gene have been reported, e.g., the exchange of glycine at pos. 54, with different impact on azole resistance in clinical isolates (Lelievre et al., 2013). Recently, a novel azole resistance mechanism was reported, which was caused by a mutation in the CCAAT-binding transcription factor (TF) complex subunit HapE (Camps et al., 2012). HapE interacts with the Hap-complex, which is, besides other functions, also important for adaptation to iron starvation and iron excess stress (Hortschansky et al., 2007).

The third and most recent class of clinically used anti-mycotic drugs are echinocandins. These molecules are composed of a cyclic hexapeptide core linked to a lipid side chain. Echinocandins act as non-competitive inhibitors of the β-1,3-glucan synthase (**Figure 1C**; Perez et al., 1983; Sawistowska-Schroder et al., 1984). Caspofungin was the first clinically applied echinocandin (CANCIDAS®, caspofungin acetate), which specifically targets the fungal CW assembly (Bowman et al., 2002). Inhibition of β-1,3-glucan synthesis results in inhibition of growth, increased osmotic sensitivity, and can even lead to lysis of cells. Besides their specificity, the use of echinocandins in clinical therapy is influenced by two drawbacks: the emergence of resistant strains, and the occurrence of the so-called paradoxical effect, which makes the drug less effective when used at high concentrations (Rocha et al., 2007; Wiederhold, 2009).

Until today, only little is known about naturally occurring resistance mechanisms against echinocandins. The most common mechanism is over-expression of the *fks1* gene in clinical isolates of *A. fumigatus* (Arendrup et al., 2009). Additionally, mutation of the Fks1 protein by substitution of a serine in position 678 by a proline (S678P) makes *A. fumigatus* resistant to caspofungin (Rocha et al., 2007). Apart from these changes, the adaptation of *A. fumigatus* to caspofungin is accompanied by a change in the CW sugar composition. As reported for other fungi, the exposition of *A. fumigatus* to caspofungin led as well to a decrease in β-glucan content and to an increase of chitin (Cowen and Steinbach, 2008; Verwer et al., 2012). In particular, these mechanisms seem to be related to the paradoxical effect exerted by this drug (Fortwendel et al., 2010). Potential mechanisms that have been suggested to induce chitin synthesis include manipulation of signaling pathways, such as the up-regulation of protein kinase C-encoding gene *pkcA*, a key component of the cell wall integrity (CWI) pathway, and elements acting in the calcineurin pathway (Wiederhold, 2007; Fortwendel et al., 2010).

## The MAPK Cell Wall Integrity Signaling Pathway in *A. fumigatus*

Several of the major findings about signaling pathways were firstly reported in the model fungus *Saccharomyces cerevisiae* (Levin, 2011). The advances in genome sequencing allowed the identification of similar or identical signaling pathways in different fungal species, revealing the presence of highly conserved proteins that can be potentially used as antifungal drug targets (Horn et al., 2012). In particular, general signaling cascades such as the mitogen activated protein kinases (MAPKs), calcineurin, cAMP, and target of rapamycin (Tor) pathways are highly conserved in the fungal kingdom (Grosse et al., 2008; Rispail et al., 2009; Baldin et al., 2015).

**FIGURE 2:**
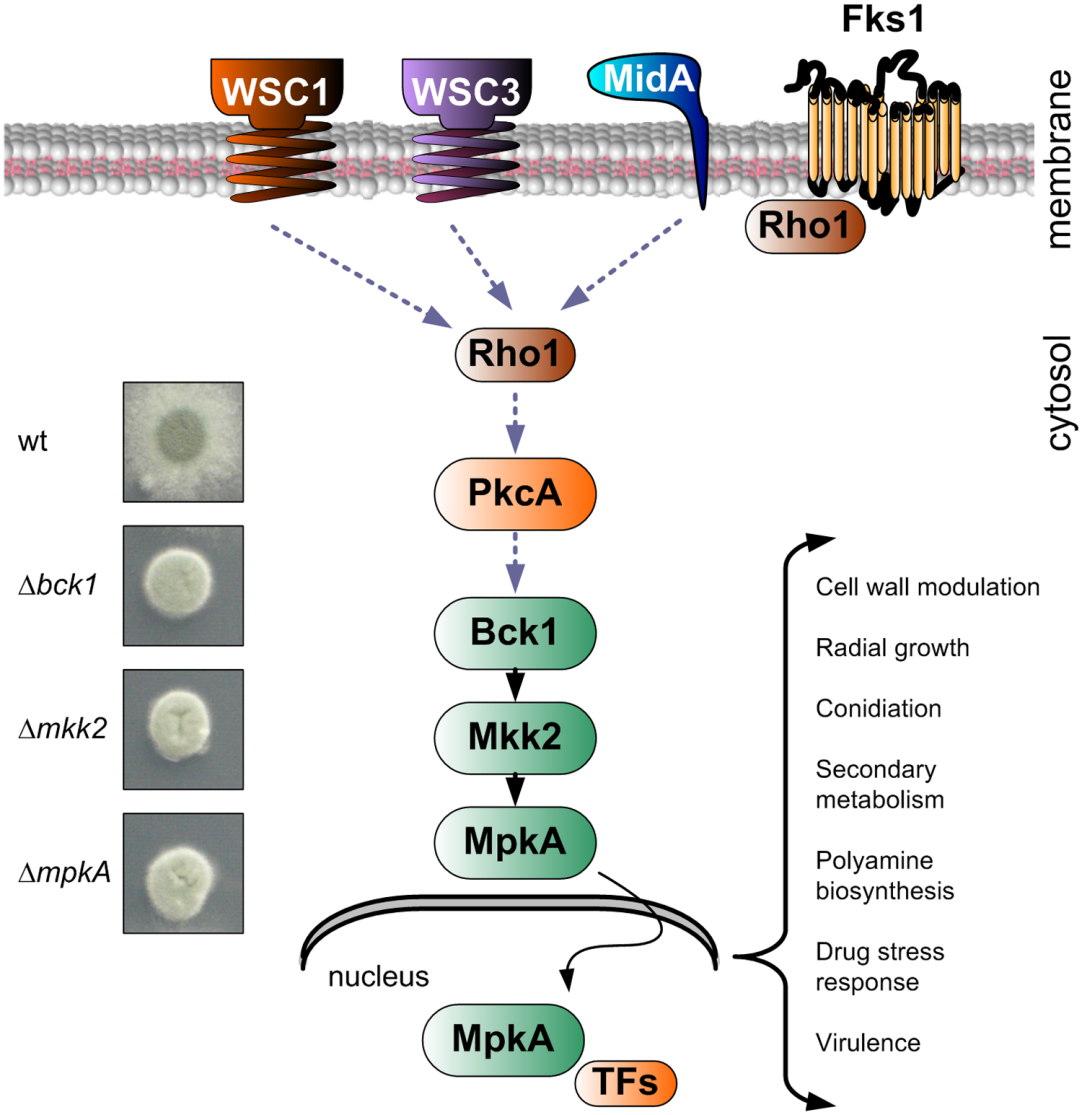
**Schematic representation of the cell wall integrity (CWI) signaling pathway in *A. fumigatus* and colony growth of indicated deletion mutants on agar plates in comparison to the wild type**. The picture shows those elements that were experimentally proven to be part of the *A. fumigatus* CWI pathway, plus some important elements, whose action was predicted based on data reported in other organisms (Fks1, Rho1, and PkcA). Once phosphorylated, MpkA translocates in the nucleus, where specific transcription factors (TFs) are activated. The deletion of genes constituting the central MAPK module have identical phenotypes, resulting in a compact colony and sensitivity to all cell wall (CW)-acting compounds (Valiante et al., 2008, 2009; Jain et al., 2011; Dichtl et al., 2012).

Among the different signaling pathways, the MAPK CWI signaling plays a major role in CW maintenance. The CWI signal pathway in *A. fumigatus* is composed of a highly conserved module formed by three MAPKs, namely Bck1 (MAPK kinase kinase), Mkk2 (MAPK kinase), and MpkA (MAPK), which sequentially phosphorylate each other (**Figure 2**; Valiante et al., 2009; Dirr et al., 2010). Upon phosphorylation, MpkA moves into the nucleus, where it likely activates transcriptional regulators (Jain et al., 2011). The deletion of one of the three kinases of the MpkA module led to the lack of MpkA phosphorylation, which is the bottleneck in the activation of the entire pathway (Valiante et al., 2008). The three mutants appear phenotypically identical (**Figure 2**).

The lack of phosphorylation of MpkA yields mutants with a typically impaired CW, showing compact colonies and reduced filamentation (Valiante et al., 2009). Additionally, mutants of all three MAPKs are equally sensitive to all known CW-disturbing compounds, which highlight their incompetence to recover from CW stress (Valiante et al., 2008, 2009).

RNA-seq analysis performed on the Δ*mpkA* mutant of *A. fumigatus* showed that the deletion of this important kinase globally changed the expression of genes putatively involved in primary metabolism. Many genes involved in sugar and amino acid metabolism were differentially regulated, whereas many well-known CW biosynthesis genes were not affected (Müller et al., 2012). This unexpected finding suggested that the CWI pathway regulates CW biosynthesis by directly affecting the synthesis of sugars, which are the essential bricks for the CW formation. As a consequence, the role of the CWI pathway seems to be not only the mere structuring of the CW, but it is likely involved in fine-tuning the balance between stress responses and energy consumption in cellular processes required for growth and development. The latter statement is strengthened by the involvement of the CWI pathway in stress responses that are not directly connected to the CW biosynthesis. As an example, MpkA was activated during iron depletion (Jain et al., 2011). When *A. fumigatus* grows under iron starvation conditions, the fungus produces secondary metabolites named siderophores, which are derived from ornithine metabolism. Siderophores are secreted to bind external iron from the environment. The production of such metabolites is rather energy-consuming (Haas, 2014). The lack of *mpkA* in *A. fumigatus* increased the global siderophore production during iron depletion by positively regulating the metabolic flux toward the ornithine metabolic pathway. Living cells use ornithine and its precursor arginine for polyamine production. The functions of these small aliphatic molecules like putrescine and spermidine are still a matter of debate, but it was shown that their concentration accompanies certain developmental transitions or exposure to stress conditions (Minguet et al., 2008). In fact, the synthesis of putrescine and spermidine has been proven to be essential for life (Chattopadhyay et al., 2002). As a result, the increased production of siderophores occurring during iron starvation conditions negatively influenced the global polyamine production and storage (Jain et al., 2011). These results suggested that during iron starvation, a functional MpkA is required to act as a central regulator to balance essential polyamine formation and *de novo* siderophore production. Additionally, the latter mechanism seems to be independent from the well-known HapX-SreA iron regulation system in *A. fumigatus* (Hortschansky et al., 2007; Gsaller et al., 2014), suggesting that the role of this kinase is epistatic to HapX-SreA in the stress response process.

Besides siderophore production, the CWI pathway seems to be an important regulator of secondary metabolism in *A. fumigatus*. Transcriptome data revealed that more than 50% of the identified secondary metabolite gene clusters were affected by the lack of *mpkA* (Müller et al., 2012). This also included the production of important virulence determinants such as gliotoxin and melanins (Jain et al., 2011). Melanins are a class of brown-pigmented molecules, which are often associated with the CW. Their main role is to protect the organisms from exogenous stresses, contributing to the first line of defense against external hazards (Heinekamp et al., 2012). *A. fumigatus* produces two kinds of melanins: the dihydroxynaphthalene (DHN) melanin, which is responsible for the characteristic gray-greenish color of the conidia (Langfelder et al., 2003), and pyomelanin, which is produced during tyrosine catabolism (Schmaler-Ripcke et al., 2009). The expression of the DHN-melanin biosynthesis gene cluster was found to be decreased in the Δ*mpkA* mutant strain. However, these mutants were still able to produce DHN-melanin, suggesting that the role of the CWI pathway in the regulation of the cluster is significant but not decisive. Concerning pyomelanin, a higher production of this compound was found in the Δ*mpkA* mutant strain (Valiante et al., 2009). These data confirmed that pyomelanin exerts a protective role, potentially compensating CW stress effects in a dysfunctional CWI background.

As summarized here, the *A. fumigatus* CWI pathway is required for many physiological states, and the CW maintenance is not its only function. It is still a matter of debate whether the CWI signaling is actively involved in the regulation of such a variety of processes or whether the observed phenotypes of corresponding deletion mutant strains are a consequence of the strong physiological impairment derived by the lack of a functional phosphorylation cascade. The meaning of the CWI signaling in *A. fumigatus* for virulence is unclear. The lack of *mpkA* did not affect virulence in a cyclophosphamide-cortisone-acetate murine infection model (Valiante et al., 2008). By contrast, the Δ*mkk2* mutant strain showing the same phenotypes as the Δ*mpkA* mutant *in vitro*, displayed reduced virulence in a murine systemic infection model (Dirr et al., 2010). These results suggest that in spite of the strongly reduced growth, the presence of neutrophil granulocytes is needed to clear the infection caused by these mutants.

Comparative analysis of signaling cascades between different fungi highlighted that the central core of signaling, i.e., kinases, are conserved, while upstream (receptors), and downstream (TFs) elements are more diverse (Rispail et al., 2009). In particular, receptor families are conserved, but vary in number. This suggests that activation of signaling is species-specific.

One of the most studied class of receptors acting upstream to the CWI signaling are the so-called WSC receptors (CWI and stress response components) (Nanduri and Tartakoff, 2001), which are characterized by the presence of highly repetitive domains containing serine and threonine residues. The WSC receptors also contain a putative carbohydrate-binding domain, which suggests that they could function as a bridge between the CW itself and the cytoplasmic membrane. Studies on the *S. cerevisiae* Wsc1 receptor suggested that these receptors work as a nanospring, able to detect/sense pulses derived from mechanical stress (Dupres et al., 2009). BLAST analysis revealed that the genome of *A. fumigatus* contains four putative genes coding for WSC receptors (*wsc1*, *wsc2*, *wsc3,* and *mid2*; Dichtl et al., 2012). All these proteins were found in the cytoplasmic membrane. However, their activity in *A. fumigatus* appears to be partially redundant, and a clearer CW phenotype was observed when multiple genes were simultaneously deleted. Additionally, the function of *wsc2* could not be related to the CWI signaling.

Other receptors putatively acting upstream of MAPK signaling pathways are the G protein coupled receptors (GPCRs). The number of these receptors also varies between different species (Grice et al., 2013). Deletion of the *gprC* and *gprD* genes in *A. fumigatus* produced mutant strains impaired in CW-related stress response (Gehrke et al., 2010).

In *S. cerevisiae*, receptors activating the MAPK signaling cascade are connected to the MAPK module *via* different signaling elements. BLAST analysis revealed that those elements are also conserved in the *A. fumigatus* genome (Rispail et al., 2009). Two of them, Rho1 and PkcA, are supposed to be essentials (Dichtl et al., 2012; De Souza et al., 2013); thus, their action should not only be related to the CWI signaling (**Figure 2**; Dichtl et al., 2010). Rho1 belongs to the Ras homolog family, which is a group of important signaling proteins (Levin, 2011). In *A. fumigatus*, Rho1 is localized in the hyphal tip, and it is misplaced by addition of farnesol (Dichtl et al., 2010), which suggests that this Ras protein interacts with putative membrane receptors to transduce external stimuli and to activate signaling cascades. A more evident phenotype was reported for the Rho guanyl-nucleotide exchange factor Rom2, which clearly localizes to the hyphal tips and septa, and its reduced expression strongly effects CW shape and stability (Samantaray et al., 2013).

Concerning PkcA (protein kinase C), there are no studies describing its function in *A. fumigatus*, but the repression of this gene in the closely related fungus *Aspergillus nidulans* affected the production of penicillin, which supports the involvement of the CWI pathway in the regulation of secondary metabolism (Herrmann et al., 2006). However, although these results suggested that the role of Rho1 and PkcA is conserved in fungi, the link between membrane receptors and the activation of the CWI pathway was shown in model yeasts, but not experimentally confirmed in *Aspergillus* species until now (Levin, 2011).

As mentioned before, the MAPK module is supposed to modulate the activity of transcriptional factors, associated with CWI signaling. Transcriptional regulators that act downstream of the CWI signaling pathway in *S. cerevisiae* were identified by BLAST analysis also in the *A. fumigatus* genome (Rispail et al., 2009; Levin, 2011), but were mostly not analyzed in detail yet. Deletion of two genes coding for putative zinc-finger TFs, named *dvrA* and *ace2*, resulted in phenotypes affecting the CW. In particular, mutants lacking *dvrA* were more resistant to nikkomycin Z, a well-known chitinase inhibitor, and more virulent compared to the wild-type strain (Ejzykowicz et al., 2010; Verwer et al., 2012). A similar phenotype was observed for the Δ*ace2* strain, which displayed abnormal pigmentation, as well as increased virulence (Ejzykowicz et al., 2009). Nonetheless, besides these phenotypes, it remains to be shown whether these TFs act downstream of the CWI signaling pathway in a MAPK-dependent manner.

## Cross Talk Between Different Signaling Pathways

*Aspergillus fumigatus* is challenged by a multitude of external stimuli, each of them needing an appropriate response. Although several different signal transduction pathways exist to initiate the required transcriptional changes, they would not be able to respond to environmental signals in a balanced way if they only acted separately in a linear manner. Therefore, an interaction between the pathways is likely. For *S. cerevisiae*, cross talk between the CWI pathway and other signal transduction pathways under different conditions have been described (Fuchs and Mylonakis, 2009), but in *A. fumigatus* this interesting and complex field of research is just emerging.

In *S. cerevisiae*, the connection between the high osmolarity glycerol (HOG) and the CWI signaling pathway has been elucidated in detail. Under CW stress, the pathways can display co-regulatory roles depending on the stress-inducing agent. It was shown that Hog1 (the yeast SakA ortholog) was activated in the absence of Slt2 (the yeast MpkA ortholog) under conditions causing CW stress (Bermejo et al., 2008; Garcia et al., 2009), indicating an inhibitory effect of the CWI signaling on the HOG pathway.

*Candida albicans* also shows the previously mentioned paradoxical effect in response to high dose exposure to caspofungin. When analyzing the response to this antifungal drug, the involvement of several signal transduction cascades was discovered, including CWI, HOG, and calcium/calmodulin-dependent calcineurin signaling, suggesting an interaction of these three pathways (Wiederhold et al., 2005; Munro et al., 2007; Walker et al., 2008). For *A. fumigatus* the participation of calcium-mediated signaling in the paradoxical growth in response to caspofungin has been described as well (Fortwendel et al., 2010). Calcium signaling involves the Ca^2+^-binding protein calmodulin and the serine/threonine protein phosphatase calcineurin (Carafoli, 2005). Deletion of the calcineurin subunit A-encoding gene *cnaA* (also named *calA*) in *A. fumigatus* resulted in the loss of the paradoxical growth phenotype under caspofungin stress, which was attributed to the transcriptional regulation of chitin synthase-encoding genes by this signal transduction pathway (Fortwendel et al., 2010). This finding shows that the caspofungin response in *A. fumigatus*, apart from CWI and HOG signaling, also involves the calcium signal transduction cascade suggesting an interaction between all three pathways in this filamentous fungus, as it was described for *C. albicans*. This conclusion was supported by the finding that in *A. nidulans* the constitutive over-expression of protein kinase C (PkcA) can in part restore the wild-type phenotype of a Δ*cnaA* deletion mutant (Colabardini et al., 2014). In addition, CnaA affects CWI signaling by regulating MpkA phosphorylation. Moreover, PkcA has an influence on the transcription of calcium-related processes as well as on the maintenance of normal intracellular calcium levels (Colabardini et al., 2014). For the basidiomycete *Cryptococcus neoformans*, an interaction of the CWI pathway and calcium signaling has also been described (Kraus et al., 2003). This suggests that the cross talk between both pathways could be common to different fungal species.

Another survey shows that not only inhibitors of the calcineurin signal transduction cascade but also rapamycin, the inhibitor of the Tor signal transduction pathway, dramatically increased the effect of caspofungin on *A. fumigatus* (Kontoyiannis et al., 2003). As another component involved in the response to caspofungin, TOR might as well interact with CWI, HOG, or calcium signaling to coordinate the response to this drug. Altogether, this model raises the question whether in *A. fumigatus* the cross talk between signaling pathways is more pronounced than it is thought today.

In the last years, several transcriptomics studies of *A. fumigatus* signal transduction mutants were published (Malavazi et al., 2009; Jain et al., 2011; Müller et al., 2012; Macheleidt et al., 2015). The generated data suggest potential interactions of the CWI signaling cascade with other signal transduction pathways. For example, in a microarray analysis of the Δ*mpkA* strain, two calcium/calmodulin dependent kinases were found to be differentially regulated in the mutant compared to the wild type under stress conditions induced by glucanex, which lyses the CW (Jain et al., 2011). Furthermore, a microarray hybridisation approach comparing the transcriptional profile of the calcineurin mutant Δ*calA* with the wild type, found the two MAP kinase kinase-encoding genes *mkk2*, involved in CWI signaling, and *pbs2*, involved in the HOG pathway, to be significantly down-regulated in the mutant strain (Malavazi et al., 2009). Data indicate once more a potential co-regulation of central signaling pathways in *A. fumigatus*. The increasing number of transcriptome analyses will lead to the identification of further interactions between signaling cascades, and most likely reveal the complexity of signal transduction in *A. fumigatus*.

## Cell Wall Impaired Mutants and Virulence

It is common praxis in infection biology to test the virulence of mutant strains in mouse infection models, in order to define whether genes have a potential function as virulence determinant. Among the different *A. fumigatus* mutant strains having a defect in the CW, more than 20 were affected in virulence (**Table 1**). Surprisingly, many mutant strains with defects in CW biosynthesis enzymes were still virulent in the applied infection models. As an example, both the α-1,3-glucan synthase mutant strains, Δ*ags1,* and Δ*ags2*, were still pathogenic, although their α-1,3-glucan content was reduced by 50% compared to the wild-type strain (Beauvais et al., 2005). Moreover, the Δ*ags3* mutant was reported to be even hyper-virulent (Maubon et al., 2006). To our knowledge, only the deletion of the genes encoding for a β-1,3-glucanosyltransferase (*gel2*) and a α-1,2-mannosyltransferase (*mnt1*) resulted in a decrease of virulence (Mouyna et al., 2005; Wagener et al., 2008).

**Table 1:** The table lists all *A. fumigatus* mutant strains that show an altered CW structure and were reported to be involved in virulence.

Accession numbers	Associate function	Name	Function related to the cell wall (CW)	Virulence of loss-of-function mutant	Reference
AFUA_1G05800	MAP kinase kinase	*mkk2*	Essential for cell CWI signaling	Decreased	Dirr et al. (2010)
AFUA_1G09280	Protein phosphatase 2C	*ptcB*	Deletion strain is more sensitive to CW-acting compounds	Decreased	Winkelstroter et al. (2015)
AFUA_1G10880	P-type calcium ATPase	*pmcA*	Gene deletion affects normal growth, CW shape conidiation, and virulence	Decreased	Dinamarco et al. (2012)
AFUA_1G14660	Methyltransferase	*laeA*	Gene deletion affects external hydrophobin layer	Decreased	Bok et al. (2005); Dagenais et al. (2010)
AFUA_1G15440	α-1,3-glucan synthase	*ags3*	Mutants showed an increased rate of germination and melanin production	Increased	Maubon et al. (2006)
AFUA_1G16950	Protein required for glycosylphosphatidylinositol (GPI)-anchor biosynthesis	*pig-a*	Required for the CWI	Decreased	Li et al. (2007)
AFUA_2G07770	Small monomeric GTPase Ras	*rasB*	Gene deletion led to irregular hyphal morphology	Decreased	Fortwendel et al. (2005)
AFUA_2G12200	cAMP-dependent protein kinase catalytic subunit 1	*pkaC1*	Important for regulation of germination, CW homeostasis, and growth	Decreased	Fuller et al. (2011)
AFUA_2G12640	G-protein coupled receptor	*gprD*	Lack of function produces CW impaired mutants	Decreased	Gehrke et al. (2010)
AFUA_2G17600	Polyketide synthase	*pksP*	Deletion blocks DHN-melanin production, production of white conidia	Decreased	Jahn et al. (1997)
AFUA_3G05650	αα-trehalose-phosphate synthase subunit TPS2	*orlA*	Gene deletion affects CWI and tolerance to high temperature	Decreased	Puttikamonkul et al. (2010)
AFUA_3G09820	C2H2 zinc finger domain protein	*dvrA*	Deletion strain is more resistant to nikkomycin Z	Increased	Ejzykowicz et al. (2010)
AFUA_3G11250	C2H2 transcription factor (TF)	*ace2*	Deletion strain displayed an abnormal conidial CW architecture	Increased	Ejzykowicz et al. (2009)
AFUA_3G12690	Putative UDP-galactopyranose mutase	*glfA*	Mutant results in a thinner CW	Decreased	Schmalhorst et al. (2008)
AFUA_4G06820	Related to sporulation-specific gene SPS2	*ecm33*	Putative glycophosphatidylinositol (GPI)-anchored CW protein	Increased	Romano et al. (2006)
AFUA_5G04170	Heat shock protein	*hsp90*	Over-expression leads to hypersensitivity to caspofungin	Decreased	Lamoth et al. (2012)
AFUA_5G08570	protein kinase A catalytic subunit 2	*pkaC2*	Important for regulation of germination, CW homeostasis, and growth	Decreased	Fuller et al. (2011)
AFUA_5G09360	Calcineurin A	*calA*	Gene deletion affects normal growth, CWI, conidiation, and virulence	Decreased	Steinbach et al. (2006)
AFUA_5G09580	Conidial hydrophobin	*rodA*	Deletion increases surface exposure of β1,3-glucan and α-mannose	Decreased	Carrion Sde et al. (2013)
AFUA_5G10760	α-1,2-mannosyltransferase	*mnt1*	Deletion leads to a higher sensitivity to calcofluor white and congo red	Decreased	Wagener et al. (2008)
AFUA_5G11230	Small monomeric GTPase Ras	*rasA*	Lack of function produces CW impaired mutants	Decreased	Fortwendel et al. (2012)
AFUA_6G10240	Sensor histidine kinase/response regulator	*fos-1 (tcsA)*	Putative histidine kinase, two-component signal transduction protein	Decreased	Clemons et al. (2002)
AFUA_6G11390	β-1,3-glucanosyltransferase	*gel2*	This gene exerts a role in conidiogenesis and CW composition	Decreased	Mouyna et al. (2005)
AFUA_7G04800	G-protein coupled receptor	*gprC*	Lack of function results in CW impaired mutants	Decreased	Gehrke et al. (2010)

As reported above, structuring of the CW depends on different signaling pathways. The observation that these pathways are connected to each other is increasingly acknowledged. Deletion of a single gene often affected more than one specific signaling pathway. In fact, many of the reported mutants with impaired CW, which displayed decreased virulence, were obtained by deleting genes putatively involved in signaling, such as Δ*calA*, Δ*rasA,* and *B*, and Δ*pkaC* mutants (Fortwendel et al., 2005, 2012; Steinbach et al., 2006; Fuller et al., 2011). Recently, it was reported that the deletion of *ptcB*, a putative protein phosphatase 2C, positively affected the phosphorylation status of both MpkA and SakA, resulting in decreased virulence (Winkelstroter et al., 2015). Similarly, blocking of CWI signaling led to reduced virulence (Dirr et al., 2010). However, blocking of CWI signaling also resulted in various physiological alterations, which are apparently not directly connected to the CW (e.g., alteration of secondary metabolism; Jain et al., 2011).

## Perspective

A major question concerns the selection of suitable and effective targets for future antifungal drugs. The three clinically important classes of antifungal drugs used against *A. fumigatus* target either enzymatic steps involved in cell membrane/CW biosynthesis or ergosterol as part of the cytoplasmic membrane. These drugs show some limitations either because they cannot really clear *A. fumigatus* infection, or because resistant strains were isolated and are therefore of major clinical concern. The increase of life-threatening mycoses accompanied with the lack of effective drugs, has fostered the search for new, broad-spectrum fungicidal agents. Drug efficacy could also be increased by the reformulation of existing antifungals as well as the search for synergistically acting compounds that can be used for treatment and prophylaxis. As an example, the effectiveness of caspofungin in prophylaxis is still a matter of debate, and there are studies indicating that caspofungin did not decrease the mortality rate of patients with diagnosed invasive aspergillosis (Karthaus, 2011). In future, because of their low toxicity, echinocandins might well be used in a combinatorial antifungal therapy with other synergistically acting drugs (Deresinski and Stevens, 2003). In line, it was already reported that the combination of caspofungin with azoles or AmB increased caspofungin activity *in vitro* (Dannaoui et al., 2004; Liu et al., 2012). Additionally, the combination of these drugs improved the efficacy of treatments of patients with severe fungal infections, in which the first line therapy failed (Nivoix et al., 2006).

The fungal CW still remains a very powerful target for antifungal drugs. However, recent studies suggest that the signaling pathways responsible for CW formation have not been completely elucidated. It remains to be shown which signaling pathways act as compensatory pathways that decrease the effectiveness of drug treatments. The identification of such pathways could lead to the discovery of new targets and new modes of action that can be exploited to potentiate the efficiency of known drugs, and to improve prophylaxis against invasive mycoses.

## Conflict of Interest Statement

The authors declare that the research was conducted in the absence of any commercial or financial relationships that could be construed as a potential conflict of interest.
